# The Effect of Furcellaran Addition and High-Pressure Homogenization Process on the Physicochemical, Rheological and Sensory Properties of Chocolate Milk Drinks

**DOI:** 10.3390/foods14223872

**Published:** 2025-11-12

**Authors:** Anita Rejdlová, Vendula Kůrová, Eva Lorencová, Zuzana Lazárková, Lucie Cmajdálková, Ludmila Zálešáková, Maciej Nastaj, Bartosz G. Sołowiej, Markéta Pětová, Tomáš Kašparovský, Richardos N. Salek

**Affiliations:** 1 Department of Food Technology, Faculty of Technology, Tomas Bata University in Zlin, Nám. T. G. Masaryka 5555, 760 01 Zlin, Czech Republiclorencova@utb.cz (E.L.); lazarkova@utb.cz (Z.L.);; 2 Department of Dairy Technology and Functional Foods, Faculty of Food Sciences and Biotechnology, University of Life Sciences in Lublin, 20-704 Lublin, Poland; maciej.nastaj@up.lublin.pl (M.N.);; 3 Department of Logistics, Faculty of Military Leadership, University of Defense, Kounicova 65, 662 10 Brno, Czech Republic; 4 Department of Biochemistry, Faculty of Science, Masaryk University, 601 77 Brno, Czech Republic

**Keywords:** chocolate milk drink, high-pressure homogenization, furcellaran, solid particle sedimentation, flow properties

## Abstract

The effect of the high-pressure homogenization (HPH) process (one-stage; applied pressure of 10 MPa and 20 MPa) and furcellaran addition (0.25% and 0.50%, *w*/*w*) on the physicochemical and rheological properties of chocolate milk drinks was evaluated. Non-homogenized samples and the milk sample used to produce all model chocolate milk drink samples were also evaluated for comparison. The addition of furcellaran and the HPH process significantly influenced the monitored characteristics of the model samples. In particular, the addition of furcellaran caused an increase in shear stress and viscosity, and HPH also had a noticeable effect on these rheological properties. From the results obtained, it can be concluded that the model samples of chocolate milk drinks exhibited a pseudoplastic behavior. Furthermore, the results of the physicochemical analyses showed a slight increase in dry matter and total soluble solids contents due to furcellaran addition. The solid particle sedimentation values of the tested samples decreased due to furcellaran addition (up to 11.99%.). In addition to the effect on rheological properties, the HPH regime slightly increased the sample’s pH values (6.80–6.81). The application of HPH and furcellaran addition may appear advantageous in manufacturing chocolate milk drinks (or dairy-based suspensions) with enhanced physical, flow and sensory properties.

## 1. Introduction

Beverages are an important part of the human diet and are among the most active categories of functional foods due to their ability to provide nutrients and bioactive substances. Milk is considered a complete food, and milk beverages account for nearly 43% of the global functional beverage market [1]. Although consumption of pure milk remains lower than that of carbonated soft drinks, consumer interest in milk beverages is growing, particularly in relation to their health benefits [2,3].

Based on production technology, milk beverages are generally classified as fermented and non-fermented. Fermented milk beverages, such as kefir, kumis, lassi and probiotic beverages, are particularly recognized for beneficial effect on human health [4]. On the other hand, non-fermented variants of milk beverages include milkshakes, flavored or enriched milks and whey-based beverages [5,6].

Flavored milk beverages represent important segment of the dairy industry, combining the nutritional benefits of milk with the organoleptic properties of added flavorings [7]. The basic ingredient of non-fermented beverages is liquid milk containing functional components that affect sensory and rheological properties. Products vary in fat content and additives such as sugar, sweeteners, and flavorings. The most popular flavors include chocolate or cocoa, vanilla, strawberry, and banana [8,9].

Cocoa contains significant amounts of bioactive compounds, notably flavonoids and methylxanthines, exhibiting antioxidant effects and associated with positive health benefits [10]. Nevertheless, its application in milk beverages may poses technological challenges. Increased cocoa content improves the sensory properties, such as flavor intensity, color, texture and viscosity; on the other hand, it also negatively affects the physical stability of the system. The low wettability and dispersibility of cocoa powder, due to the presence of fat, is particularly problematic. This results in undesirable destabilizing factors, including particle sedimentation, flake formation, and the creation of layers with different colors [7,8,10].

The addition of hydrocolloids is essential for improving the stability of cocoa-based milk beverages, as they increase system viscosity, slow sedimentation and enable the uniform dispersion of solid particles. The mechanism involves the binding of hydrocolloids to milk proteins, forming a spatial matrix that can “trap” cocoa particles, thereby reducing or even preventing sedimentation [8,10,11]. The most frequently used hydrocolloids include κ-carrageenan, sodium alginate, guar gum, xanthan gum, pectin, carboxymethyl cellulose, and inulin [8,12,13,14,15]. However, furcellaran can also be utilised in the dairy industry for the production of milkshakes and milk drinks [16,17]. Furcellaran is a sulphated anionic polysaccharide derived from the red algae *Furcellaria lumbricalis*, with structural and functional properties similar to agar and κ-carrageenan. Furthermore, furcellaran is approved in conjunction with carrageenan for use as food additives under European Union legislation [18,19].

Consumer trends increasingly favor products with minimal use of additives, prompting the dairy industry to adapt its production practices accordingly. Cocoa powders used in the production of chocolate milk beverages may contain a higher proportion of fat, which results in low solubility and a higher content of sediment in the beverages [20]. High-pressure homogenization (HPH) is considered one of the most important methods for enhancing solubility, foaming, gelling, and emulsifying abilities of functional ingredients, as well as reducing particle size. Furthermore, HPH is a non-thermal processing technology employed across diverse sectors, including chemical, pharmaceutical, biotechnological, and food industries [21,22,23,24,25]. In particular, HPH is physical process in which liquid systems are subjected to extremely high pressures (typically 10–500 MPa) and forced through a narrow valve or capillary. Under these conditions, several disruptive forces act simultaneously, including intense shear stress, turbulence, cavitation, and particle collisions. These combined effects lead to a substantial reduction in particle size, thereby improving the solubility of functional ingredients, promoting uniform dispersion, and enhancing emulsion stability. In addition, the extreme mechanical stress may contribute to microbial inactivation, further increasing the safety and shelf-life of treated products [26].

Both HPH and hydrocolloid addition have been individually studied in the context of dairy processing; however, limited information is available on combined application in chocolate milk drinks. Understanding their effect could provide new opportunities for developing products with improved techno-functional properties. Therefore, the objective of this study was to evaluate the influence of the HPH process and the addition of furcellaran on the physicochemical and rheological properties of chocolate milk drinks. By investigating these factors, the research aimed to contribute to the development of high-quality, stable, and consumer-appealing dairy-based drinks.

## 2. Materials and Methods

### 2.1. Materials

Chocolate milk drinks were prepared using the following ingredients: pasteurized milk (3.5% *w*/*w* fat content; Mlékárna Hlinsko a.s., Hlinsko, Czech Republic); alkalized cocoa powder (Gerkens^®^ GT-78 10/12%; 10–12% *w*/*w* fat content, moisture content max. 4.5% *w*/*w*—information provided by the manufacturer; Cargill cocoa & chocolate, Minneapolis, MN, USA); sucrose (Cukrovar Vrbátky a.s., Vrbátky, Czech Republic); Furcellaran Estgel 1000 (M_w_ 2.55 × 105 Da; Est-agar a.s, Kärla, Estonia).

### 2.2. Manufacture of Chocolate Milk Drinks

The chocolate milk drinks were manufactured by mixing milk, sucrose, cocoa powder, and the specified quantity of furcellaran utilizing a Stephan UMC-5 (Stephan Machinery GmbH, Hameln, Germany) apparatus. The overall preparation time was 30 min, during which the mixture was heated to a target temperature of 65 ± 1 °C and constantly stirred at 3000 rpm, to pasteurize the product and activate the furcellaran. Thereafter, the samples were homogenized using the PandaPLUS 2000 (GEA Niro Soavi, Parma, Italy) equipment. The HPH process employed a 2-L stainless steel jacketed vessel connected to a water bath (for temperature control), through which the chocolate milk drink samples were introduced into the homogenizer. In particular, single-stage HPH was performed at either 10 ± 1 MPa or 20 ± 1 MPa at 60 ± 1 °C. The samples outlet temperature after the HPH process was 63 ± 1 °C. Subsequently, the chocolate milk drinks were transferred into 0.33 L glass containers for storage (at 6 ± 2 °C) for 7 days prior to the analyses. Samples which were not exposed to HPH treatment were also manufactured. The developed samples were coded as follows: M—control sample (milk only); MC—chocolate milk, without furcellaran addition or HPH application; MC_100 and MC_200—chocolate milk samples subjected to HPH at 10 and 20 MPa, respectively. Samples containing furcellaran were also based on chocolate milk and were prepared at two concentrations: 0.25% and 0.50% (*w*/*w*). The samples MF_0.25, MF_0.25_100, and MF_0.25_200 contained 0.25% (*w*/*w*) furcellaran and were either untreated or processed using HPH at 10 or 20 MPa. Similarly, MF_0.50, MF_0.50_100, and MF_0.50_200 contained 0.50% (*w*/*w*) furcellaran, with the same HPH treatment conditions applied. The applied HPH process regimes and composition of the used ingredients is presented in Table 1.

### 2.3. Physicochemical Analysis

#### 2.3.1. pH and Dry Matter Content Determination of the Chocolate Milk Drinks

The dry matter (DM) content of the chocolate milk drinks was gravimetrically determined according to ISO 6731:2010 [27]. The pH of the chocolate mild drink samples was determined using a Foodcare pH meter (HI-99161, Hanna Instruments Inc., Woonsocket, RI, USA) with a combined glass tip electrode at 20 ± 1 °C.

#### 2.3.2. Total Soluble Solids

Total soluble solids (TSS; °Bx) were determined using a digital refractometer (Kern OTSS 45BE, Kern & Sohn GmbH, Balingen, Germany). The measurements were carried out at 20 ± 2 °C.

#### 2.3.3. Solid Particle Sedimentation

The analysis was performed according to Öztürk et al. [7]. During solid particle sedimentation analysis, samples underwent centrifugation at 6000 rpm for 20 min using an EBA 21 centrifuge (Hettich, Tuttlingen, Germany). Following centrifugation, the supernatant was carefully removed, and the remaining sediment weighed. Solid particle sedimentation of the samples was then calculated according to Equation (1).

(1) $$S=\frac{m_{1}}{m_{0}}\cdot 100$$

S Solid particle sedimentation (rel. %); $m_{1}$ weight of sediment (g); $m_{0}$ weight of sample (g).

### 2.4. Rheological Analysis

Rheological measurements were performed using a HAAKE RheoStress 1 rheometer (Thermo Fisher Scientific Brno s.r.o., Prague, Czech Republic) equipped with a concentric cylinder geometry and a fixed gap width of 2.1 mm. Each analysis involved 10 mL of sample maintained at a controlled temperature of 20.0 ± 0.1 °C. Steady-state flow behavior was assessed across a shear rate range of 0 to 150 s^−1^. Flow curves underwent nonlinear regression analysis using the Power Law model (Equation (1)) and the Herschel–Bulkley model (Equation (2)).

(2) $$\tau {=K\dot{\gamma }}^{n}$$

τ shear stress (Pa); K flow consistency index (Pa·s); $\dot{\gamma }$ shear rate (s^−1^); n Power Law index (dimensionless)

(3) $$\tau {=\tau_{0}+K\dot{\gamma }}^{n}$$

τ shear stress (Pa); $\tau_{0}$ yield stress (Pa); K flow consistency index (Pa·s); $\dot{\gamma }$ shear rate (s^−1^); n flow index (dimensionless) (Coutinho et al. [28]).

### 2.5. Sensory Analysis

The samples were assessed based on sensory attributes, including appearance, flavor, thickness, powderiness (perceived powdery sensation), and off-flavors. A panel of 14 trained assessors, comprising 10 women and 4 men aged between 21 and 54 years, participated in the evaluation. Each chocolate milk drink sample was served in a 100 mL glass container, labeled with a three-digit code, and presented in random order at a consistent temperature of 20 ± 2 °C. The sensory analysis was conducted in a laboratory equipped with individual booths for each panelist (ISO 8589) [29]. Water and white bread were provided to cleanse the palate between samples, and a 5-min break was observed after each sample testing to prevent fatigue of the palate. The attributes were rated using a 5-point scale (1—unacceptable, 3—good, 5—excellent), with each point clearly defined by specific quality criteria. Sensory analysis was conducted two times. Furthermore, no ethical approval was required for this study. Participants were informed about the study’s aim and that their participation was entirely voluntary, so that they could stop the analysis at any point and the responses would be anonymous.

### 2.6. Statistical Analysis

Each chocolate milk drink sample’s properties (physicochemical and rheological) were assessed in a minimum of 9 times (n = 9). The data obtained were evaluated by analysis of variance (one-factor ANOVA) and subsequent post-test (Tukey’s test) with 95% reliability. The sensory properties of the samples were evaluated using the Kruskal–Wallis and Wilcoxon tests. The significance level used in the tests was 0.05. Statistical analyses were performed using Minitab^®^16 software (Minitab^®^, Ltd., Coventry, UK).

## 3. Results and Discussion

### 3.1. Psychicochemical Analysis

The results of the physicochemical analyses are shown in Table 2. The dry matter content of the chocolate milk drink sample M_C and the chocolate milk drink samples with furcellaran addition (MF_0.25 and MF_0.50) was significantly higher than that of the control sample (M) (*p* < 0.05). The dry matter content of the control sample M was 12.96% (*w*/*w*), corresponding to typical values for the composition of cow’s milk [30]. Additionally, the addition of cocoa powder and furcellaran affected the dry matter content, with a slight increase in dry matter content as the amount of furcellaran added to the samples increased. However, the differences between furcellaran concentrations were not significant (*p* ≥ 0.05). Similar findings were reported also for the samples that were subjected to the HPH process.

The pH of the model samples ranged from 6.71 to 6.82. No significant differences (*p* ≥ 0.05) were observed in the tested chocolate milk drink M_C and samples with added furcellaran (MF_0.25 and MF_0.50), indicating that the addition of cocoa powder or furcellaran had no significant effect on the acid-base balance of the system (*p* ≥ 0.05). With increasing HPH pressure, a minor increase in the pH value of the samples was observed (*p* ≥ 0.05), which corresponds to the findings of studies by Gul et al. [31] and Bernat et al. [32].

**Figure 1:**
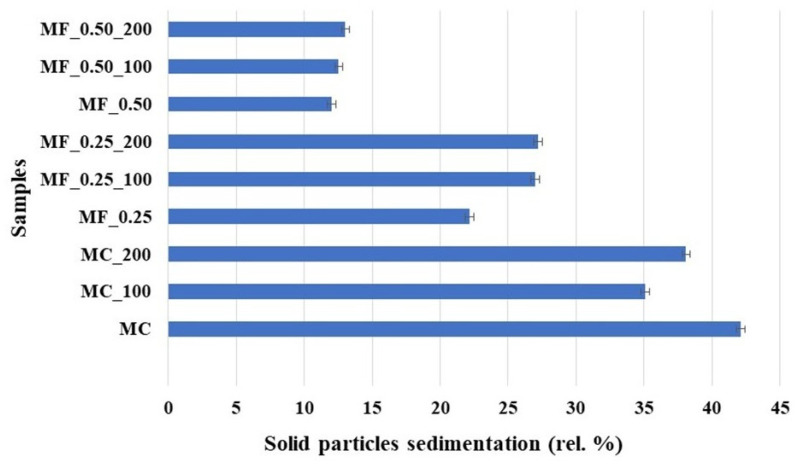
Sedimentation (rel. %) results of the chocolate milk drinks. M milk (control sample); MC chocolate milk drink; MC_100 chocolate milk drink subjected to HPH at 10 MPa; MC_200 chocolate milk drink subjected to HPH at 20 MPa; MF_0.25 chocolate milk drink contains 0.25% (*w*/*w*) furcellaran; MF_0.25_100 chocolate milk drink contains 0.25% (*w*/*w*) furcellaran subjected to HPH at 10 MPa; MF_0.25_200 chocolate milk drink contains 0.25% (*w*/*w*) furcellaran subjected to HPH at 20 MPa; MF_0.50 chocolate milk drink 0.50% (*w*/*w*) furcellaran; MF_0.50_100 chocolate milk drink contains 0.50% (*w*/*w*) furcellaran subjected to HPH at 10 MPa; MF_0.50_200 chocolate milk drink contains 0.50% (*w*/*w*) furcellaran subjected to HPH at 20 MPa. The results are expressed as means (columns) and standard deviations (bars).

Moreover, sedimentation results (Figure 1) showed significant differences between the tested samples (*p* < 0.05). The highest sedimentation value was reported for the chocolate milk drink without furcellaran and HPH use (M_C) at 42.12%, indicating higher sedimentation. From the obtained results it can be seen that the addition of furcellaran stabilized the samples. In particular, in the samples with 0.25% (*w*/*w*) added furcellaran (MF_0.25) sedimentation decreased to 22.14% and in the samples with 0.50% (*w*/*w*) added furcellaran (MF_0.50) decreased up to 11.99%. Similar results were previously reported by Rad et al. [33], where the addition of inulin to chocolate milk reduced sediment content of model sample. Furcellaran, similarly like κ-carrageenan, can reduce sedimentation in chocolate milk drink through a combination of viscosity enhancement (reported in Section 3.2), particle stabilization, and network formation. When added to milk, furcellaran can interact with calcium ions and casein micelles to form a weak and thermoreversible gel network that can entrap cocoa particles, thus preventing their sedimentation. Moreover, this formed network increases the viscosity of the continuous phase, resulting in decreased rate of particle sedimentation. Furthermore, the negatively charged sulfate groups on furcellaran molecules can adsorb onto cocoa particles, imparting electrostatic repulsion that inhibits aggregation and flocculation. These mechanisms are reinforced by electrostatic interactions between the sulphated groups of furcellaran and the amino groups of κ-casein, contributing to a crosslinked structure stabilizing the suspension [34]. HPH significantly affects the sedimentation values of tested chocolate milk drinks (*p* < 0.05). In particular, increasing homogenization pressure from 10 MPa to 20 MPa led to lower sedimentation values (Figure 1). Furthermore, in samples without furcellaran (M_C), sedimentation was the highest (42.12 rel. %). When HPH process was applied (MC_100 and MC_200), sedimentation decreased, depicting that HPH alone can improve dispersion of cocoa particles by reducing their size and promoting a more uniform distribution in the continuous phase. Additionally, when furcellaran was used, the combined effect of HPH process and hydrocolloid addition further reduced sedimentation values. The samples with 0.25% (*w*/*w*) and 0.50% (*w*/*w*) furcellaran and subjected to HPH (20 MPa; MF_0.25_200 and MF_0.50_200) showed the lowest sedimentation values, demonstrating that higher pressure enhances the stabilizing effect formed by furcellaran and milk proteins. According to Gul et al. [31], with increasing HPH pressure, sample sedimentation decreased; on the other hand, at a pressure of 150 MPa, increased sedimentation of solid particles occurred, caused probably by reduced protein solubility.

The TSS content in the model samples ranged from 20.75 to 23.79 (°Bx), which was higher than in the study by Della Lucia et al. [35], who reported TSS values ranging from 14.0 to 19.0 (°Bx). The analysis revealed a progressive increase in TSS values across all investigated chocolate milk drink samples, which could be attributed to both furcellaran addition and the application of HPH regimes. Specifically, the chocolate milk drink sample M_C exhibited the lowest TSS value (~20.5 °Bx), whereas samples with furcellaran addition and subjected to higher homogenization pressures reached values approaching 24 °Bx. This observed increase in TSS can be primarily ascribed to the addition of furcellaran, which directly contributes to the total solids content of the system [36]. Moreover, the application of HPH further increased the TSS values, which was also observed in the study by Gul et al. [31]. Increasing the homogenization pressure resulted in a modest increase in the TSS values of the model samples.

### 3.2. Rheological Analysis

The rheological parameters obtained from fitting the experimental flow data to the Power Law and Herschel–Bulkley models are presented in Table 3. In general, across all samples, high coefficients of determination (R^2^ > 0.98) indicated an excellent fit of both models to the experimental data. In the Power Law model, the consistency index (K) increased markedly with the addition of furcellaran and the application of high-pressure homogenization (HPH). The control milk sample (M) and chocolate milk drink sample M_C exhibited low K values (0.002–0.004), consistent with a Newtonian-like flow behavior. In contrast, samples containing furcellaran showed significantly higher K values, particularly at higher concentrations and homogenization pressures (*p* < 0.05). In particular, the sample M_F_0.50_200, with 0.5% *w*/*w* added furcellaran and homogenized at 20 MPa, reported a K value of 4.072, highlighting the substantial viscosity enhancement due to polysaccharide incorporation and HPH processing [37,38]. Simultaneously, the flow behaviour index (n) decreased with increasing furcellaran content and HPH intensity, indicating a more intensive shear-thinning behavior. The lowest n values (0.33–0.45) were observed in samples with 0.5% *w*/*w* furcellaran treated at 10 and 20 MPa. These results appear to be similar to that reported in the study of Gul et al. [31]. However, the values of samples without furcellaran addition were close to 1, which is comparable to the results of the studies by Codina-Torrella et al. [39] and Bernat et al. [32].

The Herschel–Bulkley model, which incorporates the yield stress (τ_0_) parameter, provided a further insight into the internal structure of the tested chocolate milk drinks. While τ_0_ values were negligible for the control sample (≤0.002 Pa), a significant increase was observed in furcellaran-added and HPH-treated samples. Notably, the M_F_0.50_200 sample exhibited a τ_0_ value of 1.7271 Pa. Moreover, the values of K and n obtained from the Herschel–Bulkley model closely mirrored those obtained from the Power Law model, but with slightly improved fitting at low shear rates, as evidenced by consistently high R^2^ values (up to 0.999). This further validates the model’s suitability for describing complex, non-Newtonian flow in such systems.

**Figure 2:**
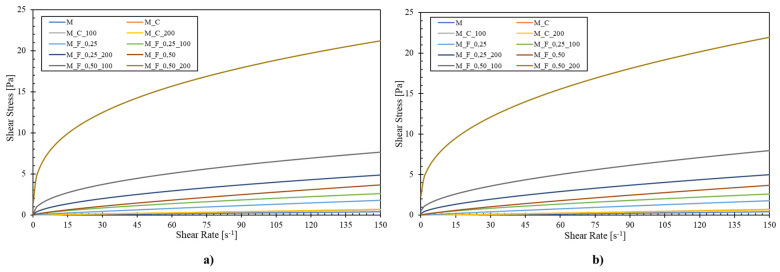
Flow curves of chocolate milk drinks modelled using (**a**) the Power Law and (**b**) the Herschel–Bulkley equations. M milk (control sample); MC chocolate milk drink; MC_100 chocolate milk drink subjected to HPH at 10 MPa; MC_200 chocolate milk drink subjected to HPH at 20 MPa; MF_0.25 chocolate milk drink contains 0.25% (*w*/*w*) furcellaran; MF_0.25_100 chocolate milk drink contains 0.25% (*w*/*w*) furcellaran subjected to HPH at 10 MPa; MF_0.25_200 chocolate milk drink contains 0.25% (*w*/*w*) furcellaran subjected to HPH at 20 MPa; MF_0.50 chocolate milk drink 0.50% (*w*/*w*) furcellaran; MF_0.50_100 chocolate milk drink contains 0.50% (*w*/*w*) furcellaran subjected to HPH at 10 MPa; MF_0.50_200 chocolate milk drink contains 0.50% (*w*/*w*) furcellaran subjected to HPH at 20 MPa.

Figure 2 presents the fitted flow curves of chocolate milk drinks using the Power-law (part a) and Herschel–Bulkley (part b) models. In both cases, the shear stress increased with increasing shear rate, confirming the non-Newtonian, shear-thinning behavior of all tested samples. Moreover, the samples containing furcellaran exhibited higher shear stress values compared to the control sample (M), indicating enhanced viscosity and improved structural integrity due to polysaccharide incorporation. The effect was more pronounced with increasing furcellaran concentration (0.25% and 0.50% *w*/*w*, respectively). Furcellaran, similarly like κ-carrageenan can increase the viscosity of milk-based systems primarily through their ability to form structured networks and interact with milk proteins. Specifically, the latter polysaccharides undergo a conformational transition from a disordered coil to an ordered helical structure in the presence of calcium ions naturally found in milk, resulting in the formation of a weak gel network enhancing the viscosity of the continuous phase. Additionally, furcellaran (and κ-carrageenan) can interact electrostatically with casein micelles (between negatively charged sulfate groups and the positively charged regions of κ-casein on the surface of the casein micelle), forming a crosslinked matrix that further contributes to the thickening effect. The latter interactions reduce the mobility of water molecules and dispersed particles, thereby increasing resistance to flow and improving product stability and mouthfeel. The extent of viscosity enhancement depends on carrageenan concentration, temperature, pH, and ionic strength of the medium [17,40]. Additionally, the samples that were exposed to HPH treatment presented higher viscosity probably by reducing particle size, leading to the development of a more uniform and stable suspension of cocoa and fat.

### 3.3. Sensory Analysis

The sensory evaluation (Table 4) of the chocolate milk drink samples, assessed across five key parameters—appearance, flavor, thickness, powderiness, and off-flavor—demonstrated significant differences in overall sensory quality. The sample MF_0.50_100 achieved the optimal score of 5 (excellent) across all evaluated attributes, indicating a highly favorable sensory profile between the panelists. In contrast, the MC sample received the lowest scores, with particularly poor ratings in powderiness (score of 1) and thickness (score of 2), suggesting suboptimal textural and mouthfeel characteristics. Additionally, off-flavor was consistently rated as excellent, indicating the absence of undesirable taste components. However, the attributes of appearance and powderiness presented significant differences between the tested samples. In particular, the obtained data suggest a clear trend; increasing the concentration of furcellaran and homogenization pressure, significantly enhance overall organoleptic profile. These findings underscore the importance of formulation optimization to achieve desirable sensory qualities in the final product.

## 4. Conclusions

The results of the physicochemical, rheological, and sensory analyses demonstrated that the use of furcellaran, as well as the application of high-pressure homogenization (HPH), had a notable impact on the properties of the tested chocolate milk drinks. A significant reduction in sedimentation values of the solid particles, coupled with an increase in system viscosity, was observed following the incorporation of furcellaran and subsequent application of HPH. This finding confirmed the stabilizing role of furcellaran, likely due to its hydrocolloid nature and ability to enhance the viscosity and suspension of dispersed cocoa particles. Moreover, these findings highlight the significant impact of both furcellaran content and HPH process on the rheological behavior of chocolate milk drinks. The observed modifications in flow parameters are relevant for the optimization of product texture, mouthfeel, and processing behavior in industrial applications. The benefit of the current study is the demonstration of the suitability of furcellaran as a natural stabilizing and thickening agent for chocolate milk drinks (or other dairy-based suspensions). In general, furcellaran addition in combination with HPH processing improved mouthfeel, provided higher viscosity, and significantly lower sedimentation was achieved. These findings bring valuable insights for the development of high-quality, stable, and dairy products that follow current trends and enhance the technological and sensory quality of the product. Although furcellaran faces limitations such as restricted availability and higher production costs due to its extraction from Furcellaria species in the Baltic Sea, these challenges are not prohibitive. Compared to common hydrocolloids like carrageenan, xanthan, or guar, furcellaran offers unique functional properties and represents a novel, underexplored ingredient for food applications. The use of furcellaran can open opportunities for innovation and further research in developing dairy products.

## Figures and Tables

**Table 1:** Applied high-pressure homogenization (HPH) process regimes and composition (% *w*/*w*) of chocolate milk drinks.

Sample *	HPH	Milk	Sugar	Cocoa Powder	Furcellaran
	(MPa)	(% *w*/*w*)	(% *w*/*w*)	(% *w*/*w*)	(% *w*/*w*)
MC	-	88.5	10	1.5	0
MC_100	10	88.5	10	1.5	0
MC_200	20	88.5	10	1.5	0
MF_0.25	-	88.25	10	1.5	0.25
MF_0.25_100	10	88.25	10	1.5	0.25
MF_0.25_200	20	88.25	10	1.5	0.25
MF_0.50	-	88.0	10	1.5	0.50
MF_0.50_100	10	88.0	10	1.5	0.50
MF_0.50_200	20	88.0	10	1.5	0.50

* MC chocolate milk drink; MC_100 chocolate milk drink subjected to HPH at 10 MPa; MC_200 chocolate milk drink subjected to HPH at 20 MPa; MF_0.25 chocolate milk drink contains 0.25% (*w*/*w*) furcellaran; MF_0.25_100 chocolate milk drink contains 0.25% (*w*/*w*) furcellaran subjected to HPH at 10 MPa; MF_0.25_200 chocolate milk drink contains 0.25% (*w*/*w*) furcellaran subjected to HPH at 20 MPa; MF_0.50 chocolate milk drink contains 0.50% (*w*/*w*) furcellaran; MF_0.50_100 chocolate milk drink contains 0.50% (*w*/*w*) furcellaran subjected to HPH at 10 MPa; MF_0.50_200 chocolate milk drink contains 0.50% (*w*/*w*) furcellaran subjected to HPH at 20 MPa.

**Table 2:** Results of dry matter content, pH, sedimentation, total soluble solids (TSS) and water activity of the tested chocolate milk drinks *, **.

Sample *	Dry Matter Content (% *w*/*w*)	pH	TSS (°Bx)
M	12.96 ± 0.06 ^a^	6.76 ± 0.01 ^a^	nd ***
M_C	23.35 ± 0.01 ^b^	6.71 ± 0.01 ^a^	20.75 ± 0.02 ^a^
MC_100	23.31 ± 0.03 ^b^	6.75 ± 0.02 ^a^	21.53 ± 0.03 ^b^
MC_200	23.37 ± 0.01 ^b^	6.77 ± 0.01 ^a^	22.02 ± 0.01 ^c^
MF_0.25	23.52 ± 0.03 ^c^	6.76 ± 0.01 ^a^	22.27 ± 0.02 ^c^
MF_0.25_100	23.55 ± 0.04 ^c^	6.80 ± 0.01 ^a^	22.61 ± 0.01 ^c^
MF_0.25_200	23.52 ± 0.02 ^c^	6.82 ± 0.01 ^a^	22.83 ± 0.02 ^c^
MF_0.50	23.68 ± 0.08 ^c^	6.79 ± 0.02 ^a^	23.05 ± 0.03 ^d^
MF_0.50_100	23.65 ± 0.01 ^c^	6.80 ± 0.01 ^a^	23.26 ± 0.01 ^d^
MF_0.50_200	23.61 ± 0.01 ^c^	6.81 ± 0.01 ^a^	23.79 ± 0.02 ^d^

* Results are expressed as mean value ± SD; MC chocolate milk drink; MC_100 chocolate milk drink subjected to HPH at 10 MPa; MC_200 chocolate milk drink subjected to HPH at 20 MPa; MF_0.25 chocolate milk drink contains 0.25% (*w*/*w*) furcellaran; MF_0.25_100 chocolate milk drink contains 0.25% (*w*/*w*) furcellaran subjected to HPH at 10 MPa; MF_0.25_200 chocolate milk drink contains 0.25% (*w*/*w*) furcellaran subjected to HPH at 20 MPa; MF_0.50 chocolate milk drink contains 0.50% (*w*/*w*) furcellaran; MF_0.50_100 chocolate milk drink contains 0.50% (*w*/*w*) furcellaran subjected to HPH at 10 MPa; MF_0.50_200 chocolate milk drink contains 0.50% (*w*/*w*) furcellaran subjected to HPH at 20 MPa. ** Mean values within a column followed by different superscript letters statistically differ (*p* < 0.05). *** nd: not determined.

**Table 3:** Power-Law and Herschel–Bulkley model parameters of the chocolate milk drinks *, **.

Power-Law Model				Herschel–Bulkley Model			
Sample *	K [Pa·s]	n [-]	R^2^ [-]	τ_0_	K [Pa·s]	n [-]	R^2^ [-]
M	0.002 ^a^	1.05 ^a^	0.999	0.0008 ^a^	0.002 ^a^	1.07 ^a^	0.999
M_C	0.003 ^b^	1.06 ^a^	0.999	0.0009 ^b^	0.003 ^a^	1.07 ^a^	0.999
M_C_100	0.003 ^b^	1.05 ^a^	0.999	0.0011 ^c^	0.003 ^a^	1.08 ^a^	0.999
M_C_200	0.004 ^c^	1.03 ^a^	0.999	0.0021 ^d^	0.003 ^a^	1.07 ^a^	0.999
M_F_0.25	0.022 ^d^	0.88 ^b^	0.999	0.0006 ^e^	0.021 ^b^	0.88 ^b^	0.999
M_F_0.25_100	0.082 ^e^	0.69 ^c^	0.996	0.0171 ^f^	0.073 ^c^	0.71 ^c^	0.998
M_F_0.25_200	0.031 ^f^	0.55 ^d^	0.995	0.1529 ^g^	0.222 ^d^	0.62 ^d^	0.999
M_F_0.50	0.079 ^h^	0.77 ^e^	0.999	0.0044 ^h^	0.075 ^e^	0.78 ^c^	0.999
M_F_0.50_100	0.814 ^i^	0.45 ^f^	0.988	0.5086 ^i^	0.482 ^f^	0.55 ^e^	0.999
M_F_0.50_200	4.072 ^j^	0.33 ^g^	0.985	1.7271 ^j^	2.538 ^g^	0.41 ^f^	0.999

* Results are expressed as mean value ± SD; MC chocolate milk drink; MC_100 chocolate milk drink subjected to HPH at 10 MPa; MC_200 chocolate milk drink subjected to HPH at 20 MPa; MF_0.25 chocolate milk drink contains 0.25% (*w*/*w*) furcellaran; MF_0.25_100 chocolate milk drink contains 0.25% (*w*/*w*) furcellaran subjected to HPH at 10 MPa; MF_0.25_200 chocolate milk drink contains 0.25% (*w*/*w*) furcellaran subjected to HPH at 20 MPa; MF_0.50 chocolate milk drink contains 0.50% (*w*/*w*) furcellaran; MF_0.50_100 chocolate milk drink contains 0.50% (*w*/*w*) furcellaran subjected to HPH at 10 MPa; MF_0.50_200 chocolate milk drink contains 0.50% (*w*/*w*) furcellaran subjected to HPH at 20 MPa. ** Mean values within a column followed by different superscript letters statistically differ (*p* < 0.05). K: consistency index; n: flow behaviour index; τ_0_: yield value.

**Table 4:** Results of the sensory analysis of the chocolate milk drink samples *^,^ **.

Sample *	Appearance	Flavor	Thickness	Powderiness	Off-Flavor
MC	2 ^a^	3 ^a^	2 ^a^	1 ^a^	5 ^a^
MC_100	4 ^a^	4 ^b^	3 ^b^	3 ^b^	5 ^a^
MC_200	3 ^b^	4 ^b^	3 ^b^	3 ^b^	5 ^a^
MF_0.25	3 ^b^	3 ^a^	3 ^b^	3 ^b^	5 ^a^
MF_0.25_100	4 ^c^	4 ^b^	4 ^c^	4 ^c^	5 ^a^
MF_0.25_200	4 ^c^	4 ^b^	5 ^d^	4 ^c^	5 ^a^
MF_0.50	4 ^c^	3 ^a^	4 ^c^	4 ^c^	5 ^a^
MF_0.50_100	5 ^d^	5 ^c^	5 ^d^	5 ^d^	5 ^a^
MF_0.50_200	5 ^d^	5 ^c^	5 ^d^	5 ^d^	5 ^a^

* Values are presented as the median; MC chocolate milk drink; MC_100 chocolate milk drink subjected to HPH at 10 MPa; MC_200 chocolate milk drink subjected to HPH at 20 MPa; MF_0.25 chocolate milk drink contains 0.25% (*w*/*w*) furcellaran; MF_0.25_100 chocolate milk drink contains 0.25% (*w*/*w*) furcellaran subjected to HPH at 10 MPa; MF_0.25_200 chocolate milk drink contains 0.25% (*w*/*w*) furcellaran subjected to HPH at 20 MPa; MF_0.50 chocolate milk drink contains 0.50% (*w*/*w*) furcellaran; MF_0.50_100 chocolate milk drink contains 0.50% (*w*/*w*) furcellaran subjected to HPH at 10 MPa; MF_0.50_200 chocolate milk drink contains 0.50% (*w*/*w*) furcellaran subjected to HPH at 20 MPa. ** Median values within a column followed by different superscript letters statistically differ (*p* < 0.05).

## Data Availability

The original contributions presented in this study are included in the article. Further inquiries can be directed to the corresponding author.
